# Retraction: Bacterial fucose-rich polysaccharide stabilizes MAPK-mediated Nrf2/Keap1 signaling by directly scavenging reactive oxygen species during hydrogen peroxide-induced apoptosis of human lung fibroblast cells

**DOI:** 10.1371/journal.pone.0362433

**Published:** 2026-11-10

**Authors:** The PLOS One Editors

Following the publication of this article, concerns were raised about results presented in Figs 4, 6, 8, 9, 11-13, and S3. Specifically,

- The following panels appear similar:
  - Fig 4 H_2_O_2_ (300µM) HFC (50 µg/ml) 24h and Fig 4 H_2_O_2_ (300µM) HFC (100 µg/ml) 6h.
- The following FACS results in Fig 6 appear more similar than would be expected from independent samples:
  - H_2_O_2_ 300 µM 3 h and H_2_O_2_ 300µM + HFC 250 µg/ml 6h
  - H_2_O_2_ 300 µM + HFC 250 µg/ml 3 h and H_2_O_2_ 300µM + HFC 250 µg/ml 12h
- The following lanes appear more similar than would be expected from independent results:
  - Fig 8B Bad lane 3 when flipped, Fig 8B Bad lane 5, and Fig 13A right Nfr2 (cytosol) lane 4.
  - Fig 9A Cytosol CytC lane 1 and Fig 12A Nucleus Nrf2 lane 1
  - Fig 9A Cytosol CytC lanes 2-3 and Fig 12A Nucleus Nrf2 lanes 3-4
  - Fig 13A p-JNK lane 1 and p-38 lane 5
  - Fig 13A p-JNK lanes 2-3 and p-38 lanes 2-3
  - Fig S3 p-p38 lane 1 and p-ERK lane 1,
  - Fig S3 p-p38 lane 2 and p-ERK lane 3
- The following panels appear to partially overlap:
  - Fig 11A active Caspase-3 of [1], Fig 5 Phospho p38 of [2, retracted in 3], and Fig 5 Phospho JNK of [2, retracted in 3]
  - The four right Fig 12 Control (0 µg/ml) panels and the four right Fig 12 H_2_O_2_ 300µM + HFC 250 µg/ml 24h panels.

The authors did not respond to these concerns.

SRC did not agree with the retraction. SS, SB, TKS, RS, RKB, BA, and AB either did not respond directly or could not be reached.

In addition to the above concerns, PLOS is aware of additional panel overlap concerns raised on PubPeer, involving the Fig 11A active Caspase-7 panel in [1] and panels presented in [4]. However, PLOS did not validate these concerns further as PLOS is unable to access [4].
